# On Small-Pox after Vaccination, as It Occurred at Haverfordwest, in the Months of October and November Last

**Published:** 1824-04

**Authors:** Walter D. Jones

**Affiliations:** Fellow of the Royal Medical Society of Edinburgh.


ART. VII.—*On Small-Pox after Vaccination, as it occurred at Haverfordwest, in the Months of October and November last.* By WALTER D. JONES, M.D. Fellow of the Royal Medical Society of Edinburgh.

VACCINATION had pretty generally held up its estimation until the year 1819, since which time several practitioners of eminence have doubted of its complete efficacy, in a number of instances, to prove itself a barrier to the inroad of the small-pox. The modified disease, thus produced after the cow-pox, appears in a very mild form, much resembling the chicken-pox; which would induce a belief, with Dr. Thompson,* that both might be induced by the same contagion, modified only by circumstances.

It is now several years since the small-pox has made its appearance in this neighbourhood, but the mortality it has lately produced, I am sorry to observe, is very considerable in persons who had not previously been vaccinated; whilst, in many others, who were supposed to be secured by that precaution, the disease appeared in a new and peculiar form, varying almost in every case in a family, from the perfect disease in the unvaccinated, to the most trivial pustular eruption, terminating on the seventh or eighth day, in the child who had previously undergone vaccination, and adhering to no regular law in the duration of either its eruptive or maturative stages. Notwithstanding the appearance of this modified disease in many instances after vaccination, the mildness of its approach, together with the very small proportion of its victims, should induce the profession at large to urge the vigorous extension of the Jennerian practice, which now, amongst the generality of people, is looked upon with great distrust.

Amongst a variety of cases which I have seen of this singular modified disease, only one has been accompanied by serious consequences; and that was in the case of a little girl, three years of age, who, after the termination of the efflorescence which frequently precedes the eruption, became suddenly and totally blind, exhibiting great dilatation of the pupils, without any other symptom, indicating an exudation of lymph in the lateral ventricles of the brain, and consequent pressure on the origin of the optic nerves. The disease, however, went on mildly, and terminated on the ninth day, when the child recovered its usual good health and vivacity; but the total blindness continued about three months. During the first part of this interval, mercury was administered to a considerable extent.

* *Edinburgh Med. and Surg. Journal,* Jan. 1819.

Blisters were applied to the head, and proper attention was paid to the state of the bowels; but without any apparent good effect. At the termination of the above period, she began gradually to recover her vision, though she has undergone no ulterior treatment. During this long period she has enjoyed excellent health.

I shall only subjoin one case out of several that I have noted down, for the purpose of marking the mildness of the disease; and, as I would hope, to increase the faith of the medical world in the utility of vaccine inoculation, which has been lately much shaken.

November 8th, *(first day.)*—F. M., aged sixteen years. She was vaccinated in March, 1809; complains of head-ache, pains in the back and limbs, thirst, and other symptoms of fever; tongue white; bowels costive; pulse 120, hard and small. Purgatur alvus.

Nov. 9th, *(second.)*—Febrile symptoms more active; heat of skin considerable; pulse 115. Habeat mistur salin. ammoniat.

Nov. 10th, *(third.)*—Symptoms continue, with a little abatement. Repet. med.

Nov. 11th, *(fourth.)*—Small red spots on the arms, neck, and face. Fever has entirely disappeared. Pulse 90, soft.

Nov. 12th, *(fifth.)*—Pustules increase in size and number; some contain matter, and are pointed; others appearing on the head and chest; appetite good.

Nov. 13th, *(sixth.)*—Pustules continue to increase in size and number; several pit in the middle. Pulse 80; complains of sore throat. Habeat gargarism.

Nov. 14th, *(seventh.)*—Pustules appear nearly stationary.

Nov. 15th, *(eighth.)*—Pustules become brown and flat; general health good.

Nov. 17th, *(tenth.)*—Pustules entirely dried up, and are of a dark-brown horny appearance; sore throat gone; no complaint.

Nov. 19th, *(twelfth.)*—Pustules fallen off, leaving a little redness behind, without any pits or other permanent marks.

Several cases, very similar to the above, might have been added; but, as the fever in this was the most severe I had seen, and the appearance of the disease more regular than in the others, perhaps it will appear more satisfactory.

There have been two cases of small-pox, within the last year, occurring *twice* in the same persons, near this place; and, if perfect immunity cannot be insured in all persons who have been inoculated for the small-pox, (and some of these have even had the disease a second time in the confluent form,) is it not safer to trust a substitute, which, in a very extensive proportion of instances, secures them permanently from a loathsome disease? while in those few (comparatively speaking,) who, from a peculiarity of constitution, do receive the infection of the small-pox after vaccination, the morbid affection thereby produced is so far altered and modified, in its appearance and consequences, as to require little or no medical treatment.

*Haverfordwest; Feb. 20th,* 1824.

